# A preliminary analysis of prevalence and associated factors of dysphagia in hospitalized elderly patients with fractures

**DOI:** 10.3389/fneur.2026.1919286

**Published:** 2026-08-27

**Authors:** Yongqi Xie, Honghu Li, Jiamin Liu, Yuheng Feng, Rong Chen, Xiaochun Li, Shaodong Xie

**Affiliations:** Department of Rehabilitation Medicine, Foshan Hospital of Traditional Chinese Medicine, The Eighth Clinical Medical College of Guangzhou University of Chinese Medicine, Foshan, China

**Keywords:** aging, dysphagia, electromyography, fracture, influencing factors

## Abstract

**Objective:**

To determine the prevalence of dysphagia in elderly inpatients with fractures and to examine factors associated with swallowing dysfunction, with particular attention to surface electromyographic (sEMG) characteristics of the infrahyoid muscle.

**Methods:**

A total of 78 elderly hospitalized patients with fractures admitted to the Department of Rehabilitation Medicine at Foshan Hospital of Traditional Chinese Medicine from January 2025 to December 2025 were included. Swallowing function and feeding status were assessed using the water swallow test and the volume viscosity swallow test. Surface electromyographic signals of the infrahyoid muscle group were collected using biofeedback rehabilitation equipment during both relaxed and dry swallowing states. Demographic and clinical data were collected, and univariate analysis along with binary logistic regression were performed to identify factors influencing dysphagia.

**Results:**

Among the 78 patients, 54 were assigned to the dysphagia group and 24 to the control group. The prevalence of dysphagia in elderly fracture rehabilitation patients was 69.2%. The majority were aged 70–79 years (50.0%), and females predominated (75.9%). During dry swallowing, the average signal of the right dry swallowing in the dysphagia group was higher than that in the control group, and the difference was statistically significant (Z = −2.515, *p* = 0.016). The intra group comparison results of the control group showed that the activity on the right side was significantly lower than that on the left side (Z = 2.571, *p* = 0.017). Univariate logistic regression analysis showed that the average surface electromyographic signal during right-side dry swallowing was an influencing factor for swallowing function in elderly hospitalized fracture patients (OR = 1.003, 95% CI: 1.001–1.006).

**Conclusion:**

Enhanced surface electromyographic signals during right-side dry swallowing may serve as an objective electrophysiological indicator for early identification of dysphagia. In clinical practice, emphasis should be placed on controlling single-bite food volume and implementing targeted screening and intervention.

## Introduction

Dysphagia refers to the clinical manifestation of reduced efficiency and safety in eating due to structural or functional impairment of the mandible, lips, tongue, soft palate, pharynx, larynx, esophagus, and other organs (1). Swallowing function in the elderly may be impaired by neurological damage, muscle degeneration, or physiological decline of swallowing organs (1). The swallowing function of normal aging may manifest as reduced saliva secretion, decreased tongue pressure, decreased sensitivity of the mouth and throat, weakened swallowing muscle strength, delayed swallowing reflex, increased pharyngeal residue, and increased penetration (2). Moreover, iatrogenic and situational factors such as surgery, anesthesia, prolonged immobilization, and polypharmacy may further compromise swallowing safety and efficiency in hospitalized older adults.

With the acceleration of global population aging, the population aged ≥60 years has reached 297 million, and those aged ≥65 years account for 217 million (3). A meta-analysis of Chinese populations showed a prevalence of dysphagia among elderly Chinese individuals of 66% (4), with an increasing trend across age groups. Studies have shown that the prevalence of dysphagia is significant in hospitalized elderly populations, with reports from abroad reaching as high as 47% (5). Although fractures themselves do not directly cause central neurological damage, the acute post-fracture state introduces multiple secondary challenges that could compromise swallowing safety. However, hospitalized elderly patients with acute fractures may represent a distinct and underappreciated high-risk group for dysphagia. Fracture pain, limited mobility, history of intubation, postoperative delirium or cognitive changes may further exacerbate the risk of swallowing decline.

More importantly, existing studies have largely focused on community-dwelling elderly populations or dysphagia caused by stroke or neurodegenerative diseases (1), with a particular lack of research specifically targeting elderly inpatients with fractures. It remains unclear whether these fracture-related stressors translate into an elevated prevalence of dysphagia in this population, and if so, what specific modifiable factors drive this risk. Some studies have suggested that underweight (6), malnutrition (7), complete dependence in basic activities of daily living (8), and denture use (9) are associated with dysphagia in community-dwelling elderly individuals.

Therefore, to clarify the prevalence and factors associated with dysphagia in hospitalized elderly patients with fractures, this study assessed swallowing function using the water swallow test (WST) and the volume-viscosity swallow test (VVST), supplemented by surface electromyography (sEMG) signals of the infrahyoid muscle. Demographic and clinical data were collected to examine their independent associations with dysphagia via binary logistic regression. This investigation aims to provide an objective basis for early screening of at-risk patients and the development of targeted interventions.

## Methods

### Ethical approval

The study was carried out in accordance with the Declaration of Helsinki and was approved by the Medical Ethics Committee of Foshan Hospital of Traditional Chinese Medicine (Approval No. KY-2024-274). All patients or their family members signed informed consent forms.

### Study design

This was a cross-sectional study. The study enrolled elderly inpatients with fractures. We collected demographic and clinical information of all participants by reviewing the hospital medical record system. We used WST as the criterion for determining swallowing difficulties, with a WST score of ≥ 3 defined as the presence of swallowing difficulties. And VVST, as a supplementary assessment tool, was mainly used in this study to further evaluate the safety and effectiveness of swallowing. According to the presence or absence of dysphagia during hospitalization, the study subjects were divided into the dysphagia group and the non-dysphagia group. Demographic and clinical data were collected, and univariate analysis along with binary logistic regression were performed to identify factors influencing dysphagia. Two trained therapists were employed to assess swallowing function. Two well-trained therapists will conduct data organization and extraction. In the process of data extraction, if there are differences, they will be judged by a third professional to ensure data accuracy.

### Participants

Elderly inpatients with fractures treated at Foshan Hospital of Traditional Chinese Medicine from January 2025 to December 2025 were selected as study subjects. The inclusion criteria were as following: (1) meeting fracture diagnostic criteria confirmed by imaging; (2) aged 65–85 years, and (3) complete clinical data. Exclusion criteria were as following: (1) history of dysphagia, motor speech disorder, or tracheostomy, (2) severe central nervous system diseases such as traumatic brain injury, stroke, subarachnoid hemorrhage, cerebral concussion, or epilepsy, and (3) severe mental disorders such as schizophrenia, depression, or bipolar disorder.

### Sample size

This study aimed to preliminarily explore influencing factors for dysphagia in hospitalized elderly fracture patients. Based on statistical experience, for multiple regression analysis, the sample size should be at least 5 to 10 times the number of independent variables. A total of 8 independent variables were included, and the calculated total sample size was at least 40 to 80 cases, which basically met the minimum requirement for exploratory regression analysis (10).

### Protocol

#### Demographic information

Demographic information included gender, age, and Body Mass Index (BMI). The clinical information was included Activities of Daily Living (ADL), Nutritional status, and fracture type (thoracolumbar fractures, pelvic fractures, multiple fractures). BMI is a simple screening tool used to assess the overall proportional relationship between weight and height. However, it does not directly measure body composition (e.g., muscle mass, fat distribution). BMI is classified as underweight ≤ 18.5kg/m^2^, normal 18.5–24.9 kkg/m^2^, overweight ≥ 25 kg/m^2^. The Barthel Index was used to assess patients’ ADL, including 10 items: feeding, bathing, grooming, dressing, bowel control, bladder control, toileting, bed-to-chair transfer, walking on level ground, and stair climbing (11). Total score ranged from 0 to 100. ADL was classified as complete independence (100), basic independence (61–99), partial dependence (41–60), and complete dependence (0–40). NRS2002 was used to evaluate the nutritional status. This scale is a simple and objective nutritional screening tool that evaluates nutritional status through dimensions such as BMI, weight change, dietary intake, and disease severity, effectively identifying patients at moderate or high risk (≥3 points) (12). We classify fracture types based on primary and secondary diagnosis. Multiple fractures refer to injuries involving multiple bones or one bone breaking in two or more locations.

### Swallowing function assessment

#### Water swallowing test

We conducted swallowing function assessment using WST and volumetric viscosity swallowing test. The WST proposed by Japanese scholar Toshio Kubota in 1982 is simple and easy to perform and can be used for risk screening (1). Swallowing function was assessed by having patients drink 30 mL of warm water while sitting, observing coughing and swallowing time. The test was classified into five situations: (1) drinking completely in one attempt without coughing or stopping, (2) drinking in two or more attempts without coughing, (3) drinking in one attempt but with coughing, (4) drinking in two or more attempts with coughing, (5) requent coughing, unable to finish. A score of 1 was considered normal, 2 suspicious, and 3–5 abnormal (13).

#### Volume viscosity swallow test

In this study, suspicious and abnormal cases were defined as dysphagia. VVST was used to assess the safety and efficacy of the swallowing process and to determine the risk of eating. During the test, patients were placed in a sitting or semi-sitting position and were given medium (10 mL) and large (20 mL) volumes of medium (thick syrup) and high (pudding) viscosity foods (14).

#### Surface electromyography

**Figure 1:**
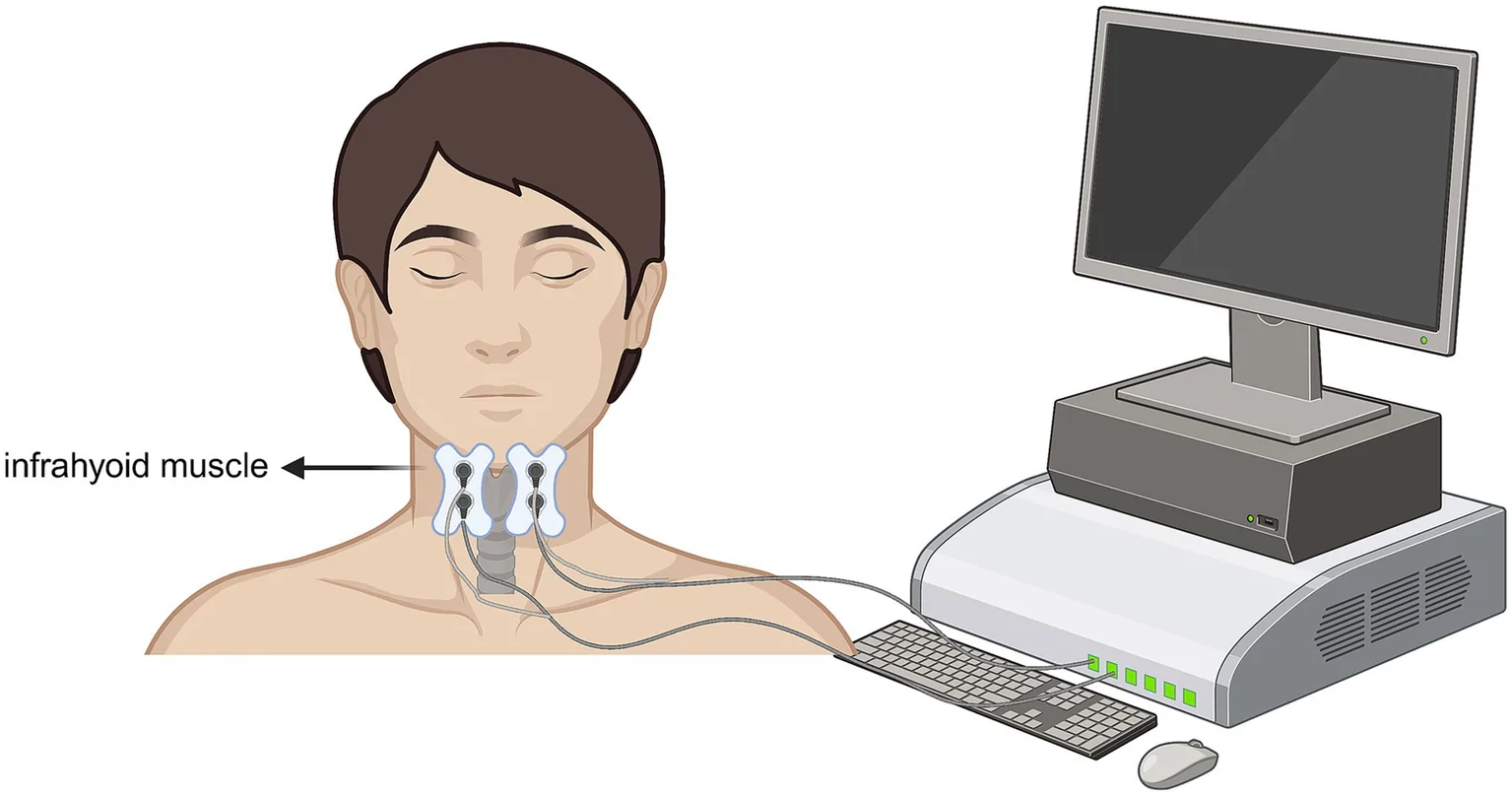
Electrode placement along bilateral infrahyoid muscles, with the lower electrode above the thyroid notch.

A biofeedback rehabilitation device (XCH-C1, China) was used to collect sEMG signals of the infrahyoid muscle group. Subjects sat upright, and signals were collected at rest and during dry swallowing (active single saliva swallow). Before signal collection, the local skin was cleaned with alcohol. Two recording electrodes were placed longitudinally along the bilateral infrahyoid muscles, with the lower edge electrode located above the thyroid cartilage notch (Figure 1). Set the scanning speed of the instrument to 2 s/D and the sensitivity to 500 uV/D. The EMG signals were transmitted via Bluetooth to a computer system for automatic processing and analysis. Changes in sEMG amplitude reflect the comprehensive electrical activity of motor unit recruitment and discharge frequency during muscle contraction, the amplitude increases with contraction intensity and can be visualized through waveforms (15).

### Statistical analysis

Statistical analysis was performed using SPSS 24.0 software. Normally distributed continuous data were expressed as mean ± standard deviation (x̅ ± s) and compared between groups using independent-sample t-tests. Non-normally distributed data were compared using the Mann–Whitney U test. Categorical data were expressed as counts (percentages) and compared using the chi-square test or Fisher’s exact test. For bilateral sEMG signals in the same patient, if the difference followed a normal distribution, a paired t-test was used; otherwise, the Wilcoxon signed-rank test was used. Taking the presence of dysphagia as the dependent variable, univariate analysis was performed using the chi-square test or nonparametric test, and variables with *p* < 0.05 were entered into binary logistic regression analysis.

## Results

From January 2025 to December 2025, a total of 122 elderly patients admitted to the department of rehabilitation medicine were screened. According to the inclusion and exclusion criteria, 44 patients were excluede for the following reasons: lumbar disk herniation (*n* = 27), shoulder surgery or frozen shoulder (*n* = 6), cervical spondylosis (*n* = 4), spinal cord injury (*n* = 3), stroke (*n* = 1), and uncooperative evaluation (*n* = 3). 78 elderly fracture rehabilitation patients were ultimately included in the study (Table 1).

**Table 1:** Comparison of demographic and clinical data between two groups of elderly fracture rehabilitation patients.

Category	Total (*n* = 78)	Dysphagia group (*n* = 54)	Control group (*n* = 24)	χ^2^/Z/t	*p*-value
Gender, n, %					
Male	22 (28.2)	13 (24.1)	9 (37.5)	1.479	0.224
Female	56 (71.8)	41 (75.9)	15 (62.5)		
Age(year), n, %	71.92 ± 5.22	72.39 ± 5.59	70.88 ± 4.26		
65–69	31 (39.7)	21 (38.9)	10 (41.7)	2.682	0.097
70–79	41 (52.6)	27 (50.0)	14 (58.3)		
80–85	6 (7.7)	6 (11.1)	0 (0.0)		
BMI (kg/m^2^), n, %	22.88 ± 3.12	22.55 ± 3.25	23.61 ± 2.74		
Too low	7 (9.0)	6 (11.1)	1 (4.2)	2.297	0.323
Normal	50 (64.1)	36 (66.7)	14 (58.3)		
Obesity	21 (26.9)	12 (22.2)	9 (37.5)		
ADL, n, %	72.44 ± 20.65	72.64 ± 21.95	73.13 ± 17.80		
Completely self-reliant	6 (7.7)	6 (11.1)	0 (0.0)	3.809	0.302
Basic self-care	47 (60.3)	30 (55.6)	17 (70.8)		
Partial self-care	18 (23.1)	12 (22.2)	6 (25.0)		
Completely unable to take care of oneself	7 (9.0)	6 (11.1)	1 (4.2)		
NRS2002	1.32 ± 0.75	1.28 ± 0.76	1.42 ± 0.72	0.756	0.452
Thoracic or lumbar spinal fracture, n, %					
No	48 (61.5)	37 (68.5)	11 (45.8)	3.613	0.057
Yes	30 (38.5)	17 (31.5)	13 (54.2)		
Hip fracture, n, %					
No	67 (85.9)	45 (83.3)	22 (91.7)	0.953	0.487
Yes	11 (14.1)	9 (16.7)	2 (8.3)		
Multiple fractures, n, %					
No	51 (65.4)	35 (64.8)	16 (66.7)	0.025	0.874
Yes	27 (34.6)	19 (35.2)	8 (33.3)		
Surface electromyography signal					
Left side relaxation average	345.06 ± 187.61	335.92 ± 192.23	365.63 ± 179.01	0.643	0.522
Right side relaxation average	340.44 ± 198.98	340.53 ± 188.94	315.61 ± 174.15	−0.550	0.584
Left swallowing average value	332.86 ± 183.75	356.80 ± 183.56	304.31 ± 229.49	−0.987	0.330
Right swallowing average value	342.79 ± 192.27	380.89 ± 173.97	258.66 ± 207.33	−2.515	0.016*

ADL, Daily living ability, NRS2002, Nutritional risk screening 2022. *p<0.05.

After screening, 54 cases of dysphagia were identified, giving a prevalence of 69.2%. The mean age was 72.39 ± 5.59 years, with the majority (50.0%) aged 70–79 years; females accounted for a high proportion (75.9%). The mean BMI was 22.55 ± 3.25 kg/m^2^, which was at the lower limit of the normal range. The mean ADL score was 72.64 ± 21.95, and the mean NRS2002 score was 1.28 ± 0.76.

Univariate analysis showed no statistically significant differences between the two groups in gender, age, BMI, ADL, NRS2002, or fracture type (thoracolumbar, multiple, or hip fractures; all *p* > 0.05), as shown in Table 1. However, the mean sEMG signal during right-side dry swallowing was significantly higher in the dysphagia group than in the control group (Z = −2.515, *p* = 0.016; Table 1). Binary logistic regression analysis showed that, compared with normal swallowing patients, the mean right-side dry swallowing sEMG signal (OR = 1.003, 95% CI = 1.001–1.006) indicated that each unit increase in the mean right-side dry swallowing signal was associated with a 0.3% increase in the odds of dysphagia (Table 2).

**Table 2:** Analysis of influencing factors on swallowing disorders in elderly fracture rehabilitation patients.

Category	B	SE	Wald	*p*-value	OR	95% CI
Right swallowing average value	0.0003	0.001	6.372	0.012*	1.003	1.001–1.006

*p < 0.05.

At rest, there were no statistically significant differences in infrahyoid sEMG signals between the two groups, nor were there significant differences between left and right sides within each group. During dry swallowing, intergroup comparison showed that the mean right-side dry swallowing signal was significantly higher in the dysphagia group than in the control group (Z = −2.515, *p* = 0.016). Intragroup comparison in the control group showed that the right-side activity was significantly lower than the left side (Z = 2.571, *p* = 0.017; Table 2).

In the 54 patients with dysphagia who underwent VVST, regardless of medium (syrup) or high (pudding) viscosity, the number of safety and efficacy events increased with increasing swallowing volume in Table 3. Among these, coughing and piecemeal swallowing occurred most frequently.

**Table 3:** Comparison of surface electromyographic signals between two groups of elderly fracture rehabilitation patients with dysphagia.

Surface electromyography signal (μV)	Dysphagia group (*n* = 54)	Control group (*n* = 24)	Between-group		Within-dysphagia group		Within-control group	
			t/Z	*p*	t/Z	*p*	t/Z	*p*
Left relaxation average	335.92 ± 192.23	365.63 ± 179.01	0.643	0.522	−0.232	0.817	1.701	0.103
Right relaxation average	340.53 ± 188.94	315.61 ± 174.15	−0.550	0.584				
Left swallowing average	356.80 ± 183.56	304.31 ± 229.49	−0.987	0.330	−1.153	0.254	2.571	0.017*
Right swallowing average	380.89 ± 173.97	258.66 ± 207.33	−2.515	0.016*				

*p < 0.05.

## Discussion

This study systematically evaluated swallowing function in hospitalized elderly patients with fractures and found that the prevalence of dysphagia was 69.2%. Although the average sEMG signal during right-side dry swallowing was statistically associated with dysphagia, the corresponding odds ratio (1.003) indicated a minimal effect size with limited clinical utility as a standalone predictor. These findings provide new clinical and electrophysiological evidence for understanding swallowing function changes in the elderly post-fracture population and have implications for early clinical identification of age-related dysphagia (Table 4).

**Table 4:** Volume-viscosity swallow test of elderly fracture rehabilitation patients with dysphagia.

Consistency	Volume (ml)	Security incidents(n)	Effectiveness event(n)
Nectar	5	9	26
	10	13	39
	20	21	61
Pudding	5	11	20
	10	14	35
	20	27	61

Safety events include coughing and changes in sound quality, while effectiveness events include lip leakage, oral residue, repeated swallowing, and pharyngeal residue.

Reported prevalence rates of dysphagia in the elderly vary widely, ranging from 30.59 to 66.0%. Lai et al. (16) performed videofluoroscopic swallowing studies on 256 elderly patients with an EAT-10 total score ≥3 and diagnosed dysphagia in 160 (30.59%). Peter et al. (17) assessed hospitalized elderly individuals using the EAT-10 and VVST and found a dysphagia prevalence of 30.7%, noting that nursing staff were rarely aware of the possibility of dysphagia in this population. Liu et al. (4) conducted a meta-analysis and found that the overall prevalence of dysphagia in hospitalized elderly Chinese individuals was 66.0%, while subgroup analysis using the 30 mL WST showed a prevalence of 35.0%. Li et al. (18) screened elderly patients with sarcopenia using the traditional WST and found that the prevalence of dysphagia increased with sarcopenia severity. The present study found a dysphagia prevalence of 69.2% in elderly fracture patients, which is higher than that in general community-dwelling elderly or general hospitalized elderly populations (17, 19). This suggests that elderly fracture patients are also prone to swallowing dysfunction, and attention to rehabilitation nursing in this area is needed. The relatively high prevalence of dysphagia in our elderly fracture patients may be related to the following three factors: first, physiological degeneration of swallowing organs (e.g., decline in oral and pharyngeal muscle function) (20, 21); second, the traditional WST requires subjects to drink 30 mL of water at once, which some researchers consider a stress test even at 20 mL (22); therefore, 30 mL poses a greater challenge to elderly swallowing function, especially requiring conscious control during the initial phase of swallowing (2). A study comparing the traditional WST with a modified water swallow test also found that stroke patients had a higher cough rate on the traditional WST (23). Third, elderly patients after fracture often experience pain and postoperative immobilization in bed, which prevents them from maintaining a safe swallowing posture (sitting or semi-sitting). However, this study did not include variables such as time to surgery or duration of postoperative immobilization for analysis. Future studies should further explore the impact of these variables on dysphagia in hospitalized elderly patients.

Logistic regression analysis found that an increased mean sEMG signal during right-side dry swallowing was statistically associated with the risk of dysphagia (OR = 1.003, 95% CI: 1.001–1.006). However, given the minimal effect size, this association should be interpreted with caution: the absolute amplitude of right-side sEMG alone appears to have limited clinical utility as a standalone predictor. Rather, the more notable finding is the qualitative difference in bilateral activation patterns between groups, which may reflect underlying neuromuscular adaptations. The phenomenon that patients with swallowing dysfunction had higher right-side EMG signals during dry swallowing than those without may be explained by the following reasons. First, compensatory effortful swallowing in the elderly. In the early stages of swallowing decline or silent aspiration, the nervous system may recruit more motor units and increase the contraction intensity of relevant muscle groups to maintain swallowing safety and efficacy, thus presenting as increased EMG signals. Peter et al. (17) found that the peak sEMG level during swallowing increased from 103 μV to 110 μV (*p* = 0.05) in patients with dysphagia, whereas no significant change was observed in those without dysphagia. Second, laterality of normal swallowing function. Studies have shown that with aging, the dominant side of swallowing tends to shift more to the left; approximately 71% of elderly individuals (aged 51–75 years) exhibit left-dominant swallowing (24). In the present study, patients without dysphagia had significantly higher left-sided infrahyoid EMG signals than right-sided signals, consistent with the above findings. However, the dysphagia group showed stronger right-sided EMG signals, which may be due to impairment or inefficiency of the normal left-sided physiological pattern, leading to overactivation of the right-sided muscle groups to maintain swallowing safety and efficacy, thereby manifesting as right-sided dominance on EMG. In addition, decreased muscle coordination may also be a cause of abnormal EMG signals. Normal efficient swallowing requires precise timing and coordination of pharyngeal and laryngeal muscle contractions. Aging or factors such as post-fracture immobilization and feeding posture may disrupt this coordination, resulting in compensatory enhancement of EMG signals. Although sEMG has limitations such as skin thickness, tissue conductivity, electrode placement, and simultaneous activity of adjacent muscles, it still provides a means for clinical insight into the physiology of the swallowing system (21). The observed bilateral asymmetry may provide more meaningful electrophysiological clues for understanding the swallowing pathophysiology of this population. Future studies with larger cohorts should investigate whether composite indices, such as right-to-left amplitude ratios or dynamic swallowing patterns, may provide greater discriminative value for early dysphagia identification than isolated unilateral measurements.

The results of the VVST provide important guidance for clinical rehabilitation and nursing of elderly fracture patients. In this study, both safety and efficacy events increased significantly with increasing single-bite swallowing volume, and safety did not improve with higher viscosity foods. This may be related to reduced saliva production in the elderly (2), where overly thick foods may actually increase swallowing difficulty and exacerbate the risk of pharyngeal residue. Peter et al. (17) also found that patients reported difficulty swallowing solid foods or pills and coughed while eating. This indicates that for elderly fracture patients, controlling single-bite volume (starting from 5 to 10 mL) is a more fundamental and critical safety strategy than adjusting food consistency. Clinically, priority should be given to “small bites and slow swallowing,” followed by individualized texture adjustments based on VVST results. In addition, special attention should be paid to the patient’s feeding posture. For patients unable to sit up due to fractures, the bed should be elevated as much as possible (≥60°) within the limits of pain tolerance and medical safety, and necessary postural support should be provided to create a relatively safe swallowing environment and reduce the risk of aspiration caused by improper posture (25).

This study did not find associations between age, gender, BMI, ADL, NRS2002, specific fracture types (thoracolumbar, hip), or multiple fractures and age-related dysphagia, which differs from some studies focusing on hospitalized elderly patients. Age is not an absolute influencing factor for swallowing dysfunction. Archer et al. (26) argued that swallowing function does not necessarily decline with age, possibly because elderly individuals can perform tasks using relative strength, and their neural drive and coordination capacity are not lost. Previous studies have shown a high prevalence of dysphagia in hip fracture patients (27), with 50.4% of elderly hip fracture patients having some degree of swallowing difficulty; factors such as female gender, postoperative confusion, and pre-admission nursing home residence were associated with the severity of dysphagia (28). The absence of an association between hip fracture and dysphagia in the present study may be due to the inclusion of multiple fracture types with varying sample sizes. The lack of association between nutritional indicators (BMI and NRS2002) and dysphagia may be because elderly individuals often have sarcopenia and redistribution of body fat, and swallowing impairment may be more related to neuromuscular control, pain, and immobilization.

## Limitations

This study has several limitations. The enrolled hospitalized elderly patients were from a rehabilitation department, and the sample size was small, which cannot represent the overall level of the regional elderly population. At the same time, the history of anesthesia and intubation related to surgery may also interfere with postoperative swallowing outcomes, and this study is limited by retrospective design and has not fully corrected for these confounding factors, which need to be considered when interpreting the results. Second, this was a cross-sectional study that cannot infer causality; whether increased right-side EMG signals are a cause or consequence of dysphagia needs to be confirmed by future longitudinal studies. SEMG is susceptible to subcutaneous fat, electrode displacement, muscle crosstalk, and other factors, which may lead to biased results. Finally, swallowing function assessment relied primarily on the WST for screening, which has low sensitivity. Future studies with available resources could combine videofluoroscopic swallowing study (the gold standard) for more precise functional staging diagnosis.

## Conclusion

The prevalence of dysphagia in hospitalized elderly patients with fractures is high. Enhanced sEMG signals of the infrahyoid muscle group during right-side dry swallowing may indicate an increased risk of dysphagia in elderly fracture rehabilitation patients. Clinical rehabilitation or nursing should pay attention to swallowing risk assessment (VVST or modified WST) in these patients and implement early intervention strategies.

## Data Availability

The original contributions presented in the study are included in the article/supplementary material, further inquiries can be directed to the corresponding author.
